# Supplementary material for: An open-source framework for large-scale, flexible evaluation of biomedical text mining systems
Source: J Biomed Discov Collab. 2008 Jan 29;3:1. doi: 10.1186/1747-5333-3-1 (PMC2276192; doi:10.1186/1747-5333-3-1)
Supplement: Additional file 1 — Evaluation platform source code. The additional file contains the software evaluation framework discussed in this paper. The most current release can be downloaded from SourceForge.net [1]. See the accompanying README file for instructions on installation and use. Also included in the distribution is a collection of UIMA wrappers for some commonly used BioNLP tools and annotated corpora. [file 1747-5333-3-1-S1.zip › bionlp_uima_repository-1.0.1/docs/api/edu/uchsc/ccp/util/nlp/KeyValuePair.html]

KeyValuePair (Center for Computational Pharmacology Util/UIMA API)


|  |  |  |
| --- | --- | --- |
| **Overview** \| **Package** \| **Class** \| **Tree** \| **Deprecated** \| **Index** \| **Help** | |  |
| **PREV CLASS**   NEXT CLASS | **FRAMES**    **NO FRAMES**     **All Classes** |  |
| SUMMARY: NESTED \| FIELD \| CONSTR \| METHOD | DETAIL: FIELD \| CONSTR \| METHOD |  |


---


## edu.uchsc.ccp.util.nlp Class KeyValuePair

```
java.lang.Object
  edu.uchsc.ccp.util.nlp.KeyValuePair
```

**All Implemented Interfaces:**: Comparable

---

``` public class KeyValuePair extends Object implements Comparable ```

This class is a utility class used for interacting with HashMaps, and potentially and application requiring Key/Value pairings.

**Author:**
:   William A. Baumgartner, Jr.

---

**Constructor Summary**

`KeyValuePair(String key,
int value)`
  
          Construct a new KeyValuePair with the given input arguments.


| **Method Summary** | |
| --- | --- |
| `int` | `compareTo(Object anotherKVP)`             This method enables comparison between two KeyValuePairs. |
| `String` | `getKey()`             Returns the key |
| `int` | `getValue()`             Returns the value |
| `void` | `setKey(String key)`             Sets the key |
| `void` | `setValue(int value)`             Sets the value |

**Methods inherited from class java.lang.Object**

`clone, equals, finalize, getClass, hashCode, notify, notifyAll, toString, wait, wait, wait`

**Constructor Detail**

### KeyValuePair

```
public KeyValuePair(String key,
                    int value)
```

:   Construct a new KeyValuePair with the given input arguments.

    **Parameters:**: `key` - the key: `value` - the value


**Method Detail**

### getKey

```
public String getKey()
```

:   Returns the key

    :   **Returns:**: a string containing the key

---


### setKey

```
public void setKey(String key)
```

:   Sets the key

    :   **Parameters:**: `key` - a string containing the key

---


### getValue

```
public int getValue()
```

:   Returns the value

    :   **Returns:**: an int value

---


### setValue

```
public void setValue(int value)
```

:   Sets the value

    :   **Parameters:**: `value` - an int value

---


### compareTo

```
public int compareTo(Object anotherKVP)
```

:   This method enables comparison between two KeyValuePairs.

    :   **Specified by:**: `compareTo` in interface `Comparable`
    :   **Parameters:**: `anotherKVP` - the KeyValuePair to compare with


---


|  |  |  |
| --- | --- | --- |
| **Overview** \| **Package** \| **Class** \| **Tree** \| **Deprecated** \| **Index** \| **Help** | |  |
| **PREV CLASS**   NEXT CLASS | **FRAMES**    **NO FRAMES**     **All Classes** |  |
| SUMMARY: NESTED \| FIELD \| CONSTR \| METHOD | DETAIL: FIELD \| CONSTR \| METHOD |  |


---
